# Supplementary material for: Context-dependent perturbations in chromatin folding and the transcriptome by cohesin and related factors
Source: Nat Commun. 2023 Sep 19;14:5647. doi: 10.1038/s41467-023-41316-4 (PMC10509244; doi:10.1038/s41467-023-41316-4)
Supplement: Supplementary file 1 — Supplementary Information [file 41467_2023_41316_MOESM1_ESM.pdf]

SUPPLEMENTAL FIGURES

Context-dependent perturbations in chromatin folding and the transcriptome by cohesin and related factors

Ryuichiro Nakato[1,§,*], Toyonori Sakata[2,3,§], Jiankang Wang[4], Luis Augusto Eijy Nagai[1], Yuya Nagaoka[1], Gina Miku Oba[1], Masashige Bando[2] and Katsuhiko Shirahige[2,3,*]

[1]Laboratory of Computational Genomics, Institute for Quantitative Biosciences, University of Tokyo, 1-1-1 Yayoi, Bunkyo-Ku, Tokyo 113-0032, Japan.

[2]Laboratory of Genome Structure and Function, Institute for Quantitative Biosciences, University of Tokyo, 1-1-1 Yayoi, Bunkyo-Ku, Tokyo 113-0032, Japan.

[3]Karolinska Institutet, Department of Biosciences and Nutrition, Biomedicum, Quarter A6, 171 77, Stockholm, Sweden.

[4]School of Biomedical Sciences, Hunan University, Changsha, China.

[§]These authors contributed equally to this work.

*To whom correspondence should be addressed: rnakato@iqb.u-tokyo.ac.jp, kshirahi@iqb.u-tokyo.ac.jp

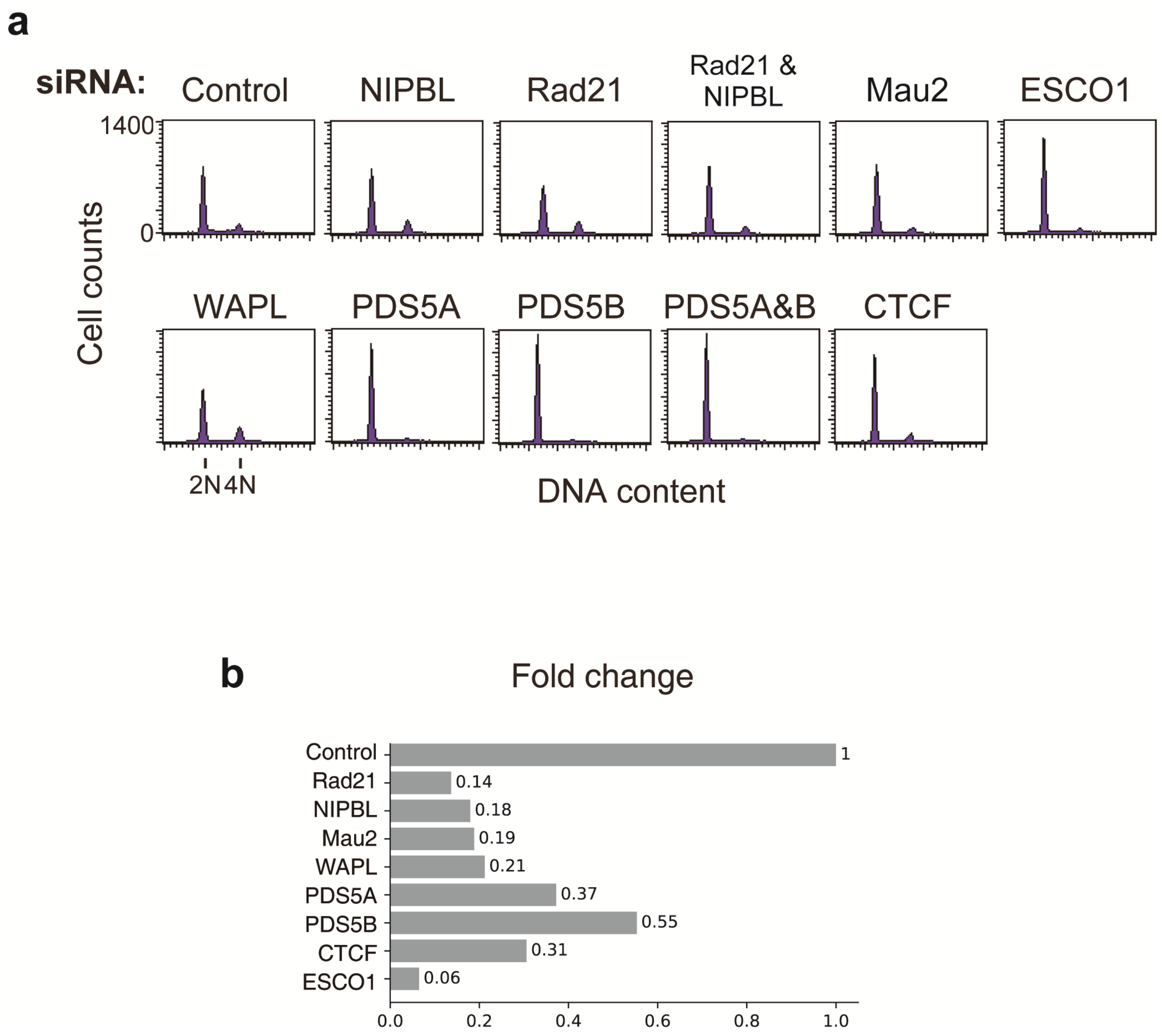

Figure S1. siRNA sample information. (a) Fluorescence-activated cell sorting plots for all depletions. Representative samples are shown. **(b)** Quantitative ratio of depletion efficiency evaluated by ImageJ (https://imagej.net/ij/) from the Immunoblots.

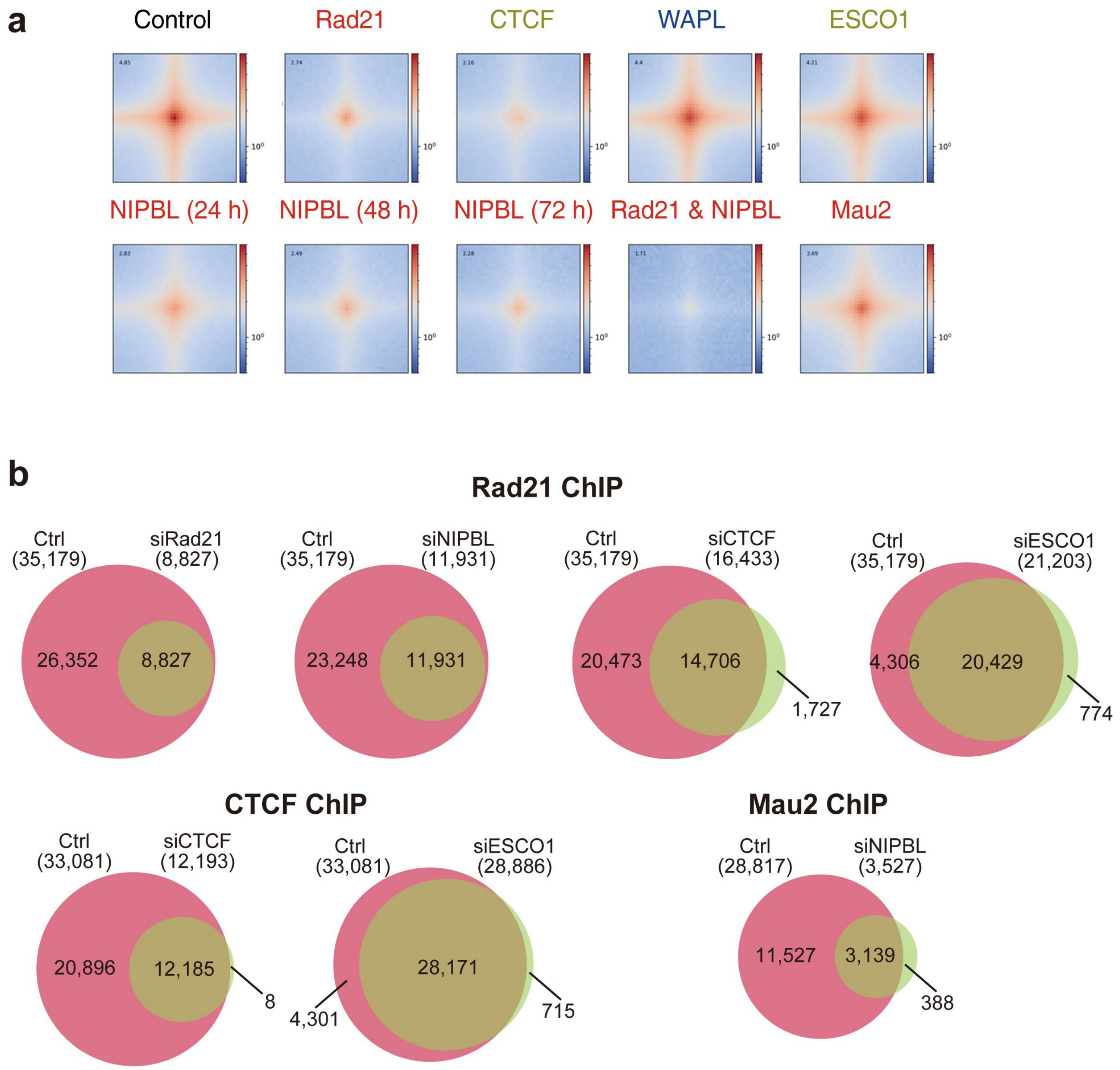

Figure S2. Depletion effects on Hi-C and ChIP-seq peaks. (a) Aggregate peak analysis (APA) of all loops in control cells and in siRNA-treated cells after Hi-C analysis. For NIPBL depletion, three different time points were assessed. In siNIPBL with the 24 h treatment (bottom left), the chromatin loop strength remained more than that with the 48 h and 72 h treatments, suggesting an insufficient depletion effect. **(b)** Peak overlap between control and siRNA-treated samples (Rad21, CTCF, and Mau2 ChIP). The numbers in parentheses indicate the number of peaks in each sample.

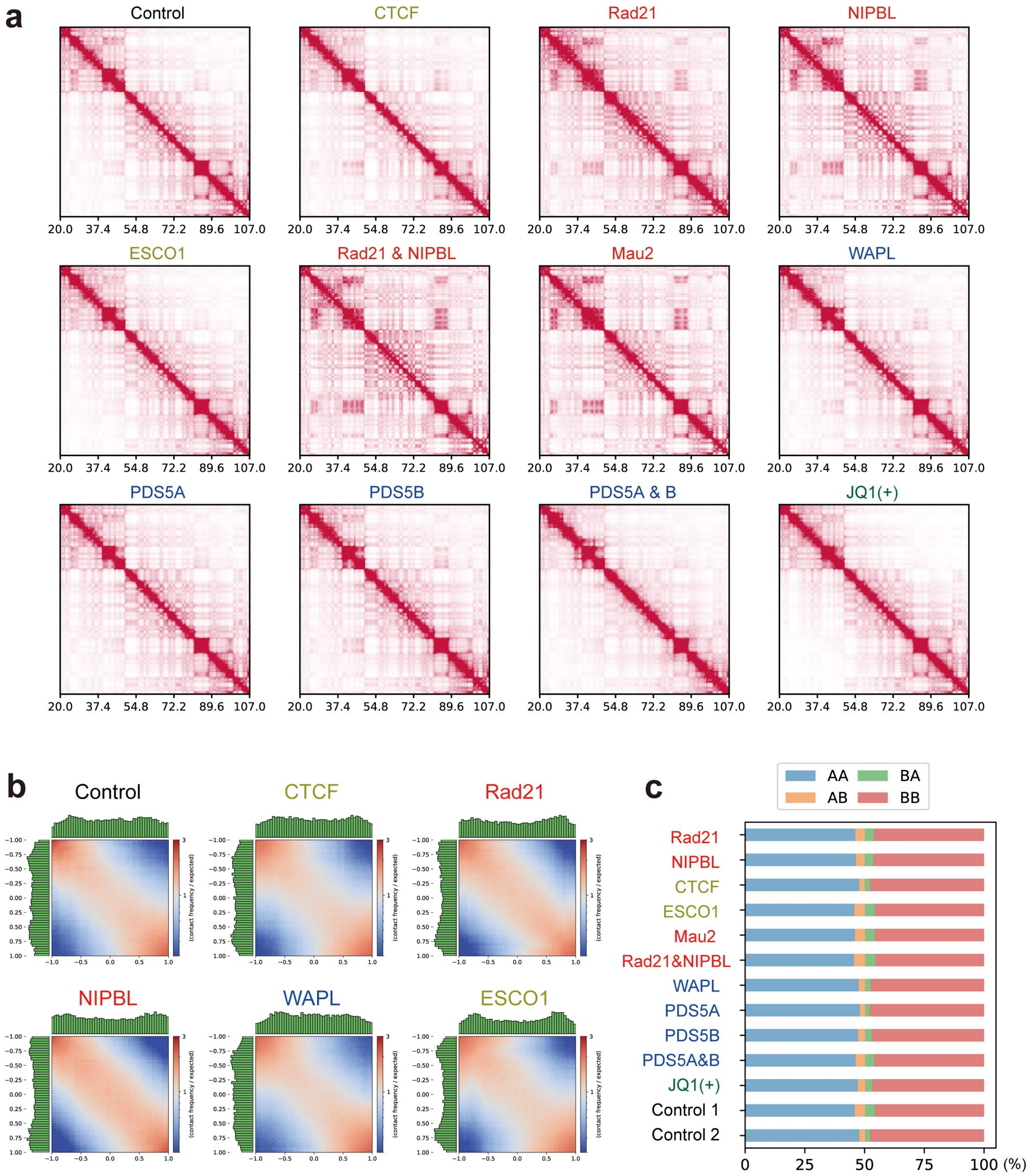

Figure S3. Compartment strength and switching. (a) Hi-C maps of a representative chromosomal region (chromosome 14, 20.0–107.0 Mb). Depletion of cohesin and loaders resulted in increases in long-range interactions within compartments A and B, visible here as a plaid (or checkerboard) pattern. **(b)** Saddle plots evaluated compartmentalization strength. Average interaction frequencies between pairs of loci were sorted by their compartment PC1 values (green histogram). **(c)** The fraction of compartment switching. A, compartment A; B, compartment B. Two control replicates are shown as negative controls.

a

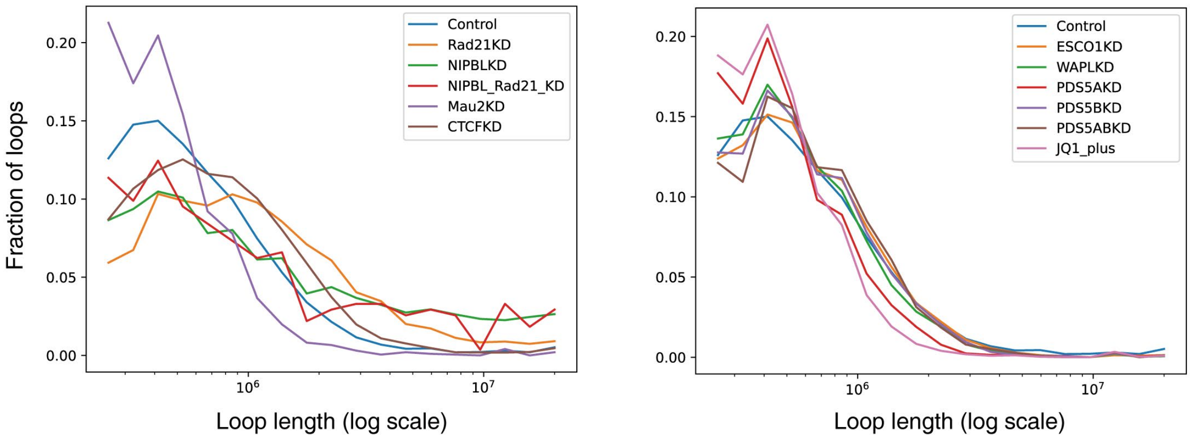
Fraction of loops
Loop length (log scale)
Loop length (log scale)
0.00
0.05
0.10
0.15
0.20
10^6
10^7
Control
Rad21KD
NIPBLKD
NIPBL_Rad21_KD
Mau2KD
CTCFKD
Control
ESCO1KD
WAPLKD
PDS5AKD
PDS5BKD
PDS5ABKD
JQ1_plus

b

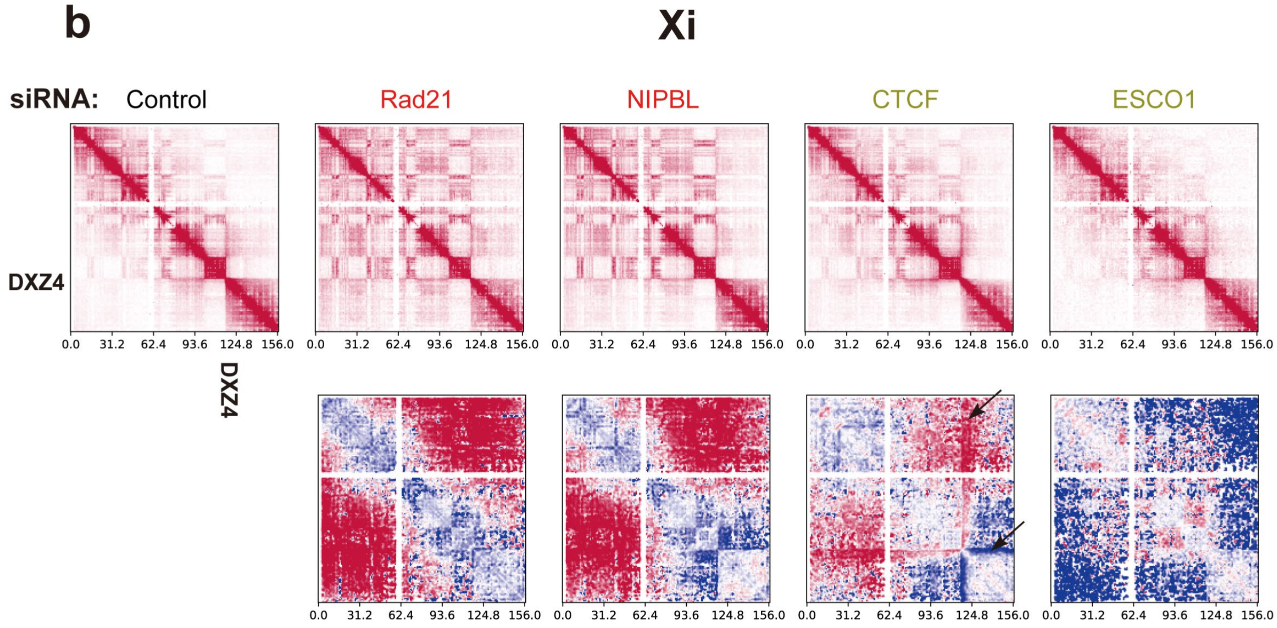
Xi
siRNA:
Control
Rad21
NIPBL
CTCF
ESCO1
DXZ4
DXZ4
0.0
31.2
62.4
93.6
124.8
156.0

c

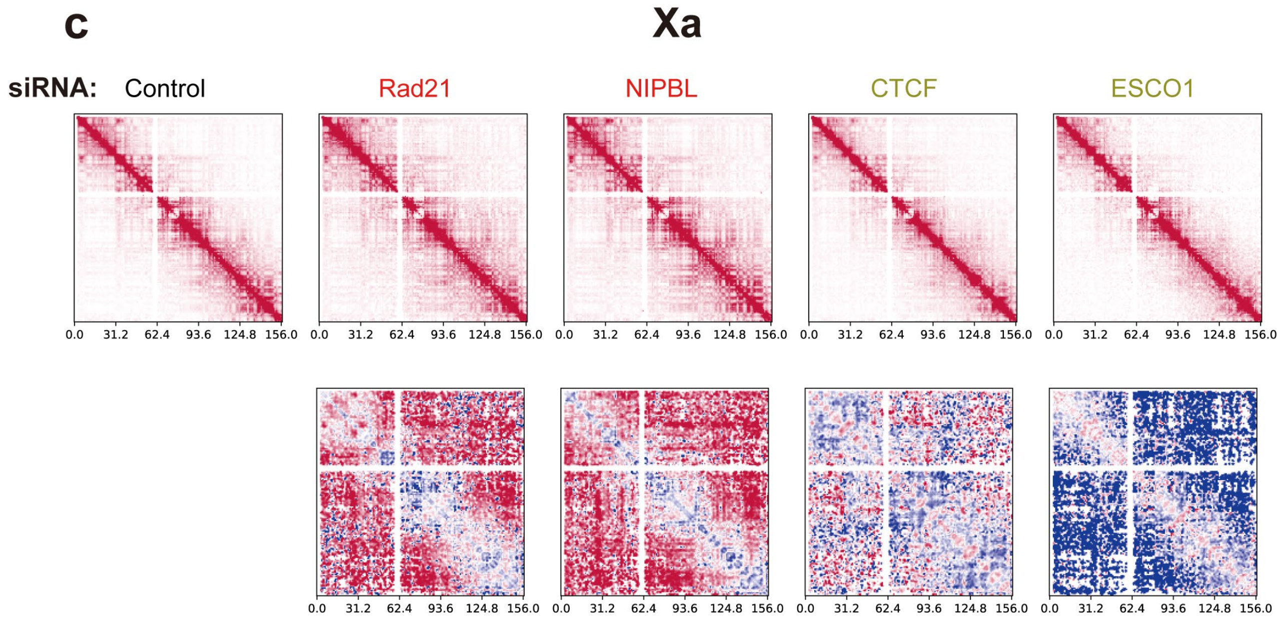
Xa
siRNA:
Control
Rad21
NIPBL
CTCF
ESCO1
0.0
31.2
62.4
93.6
124.8
156.0

Figure S4. Loop length distribution and depletion effect on active and inactive chromosome X. (a) Loop length distribution corresponding to Figure 3d. **(b, c)** Allele-specific Hi-C analysis of **(b)** inactive chromosome X (Xi) and **(c)** active chromosome X (Xa). The microsatellite repeat DXZ4 indicates the boundary of the two megadomains. Top: Normalized heatmaps. Bottom: Relative enrichment of the interaction frequency (log scale) relative to control. In Xi **(b)**, the interaction within the smaller megadomain (lower right of the heatmaps) was less affected in all samples.

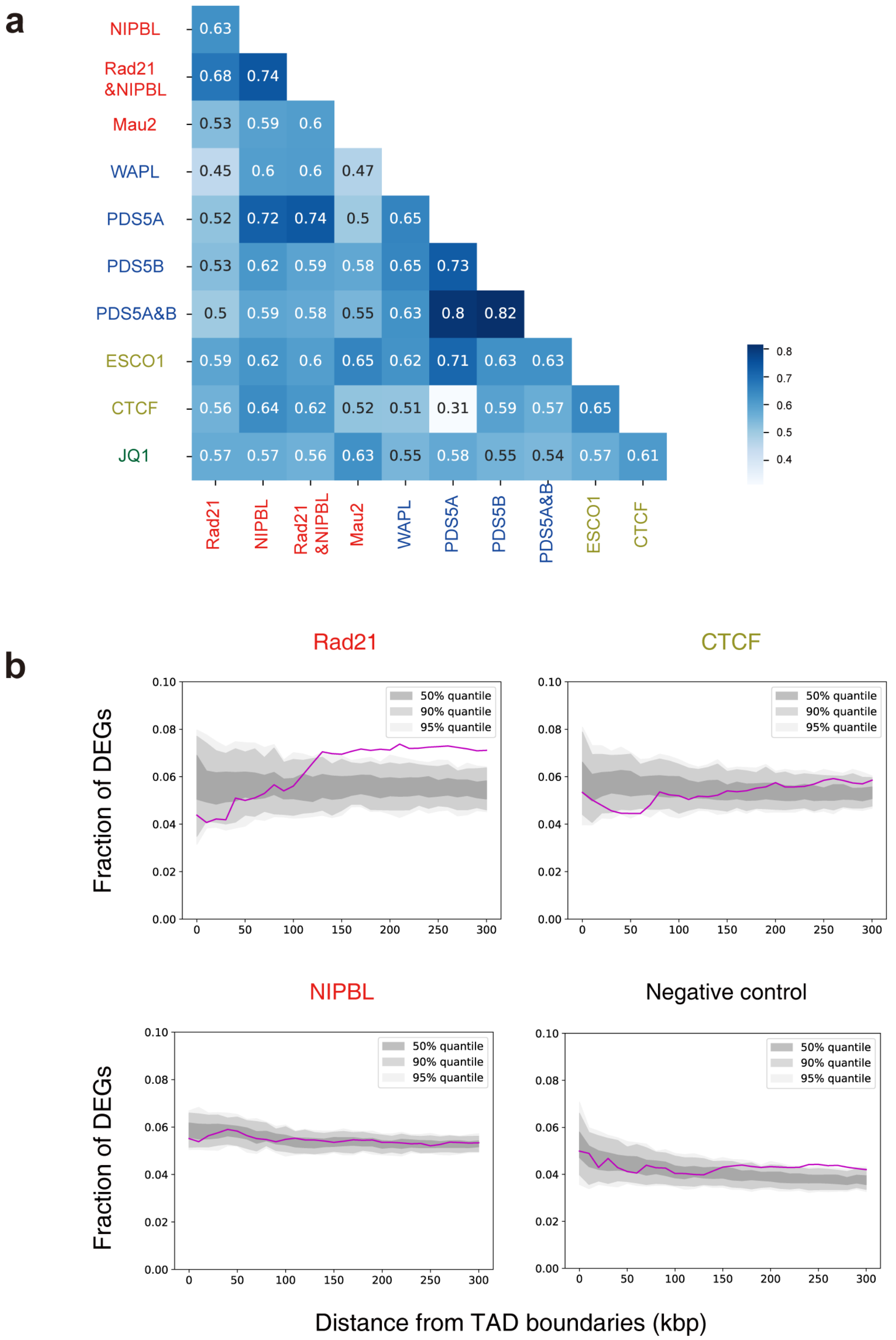

Figure S5. Comparative analysis of siRNA effects on the transcriptome. (a) Correlation heatmap based on Simpson index (overlap of all DEGs, FDR < 0.01) for all sample pairs. **(b)** The proximity of DEGs to disrupted TAD boundaries for siRad2, siCTCF, siNIPBL, and the negative control (DEGs from siMau2 against disrupted boundaries from siCTCF). Purple lines indicate the fraction of DEGs at varying distances from disrupted TAD boundaries. Gray ribbons indicate the 50, 90, and 95% percentiles from random boundaries.

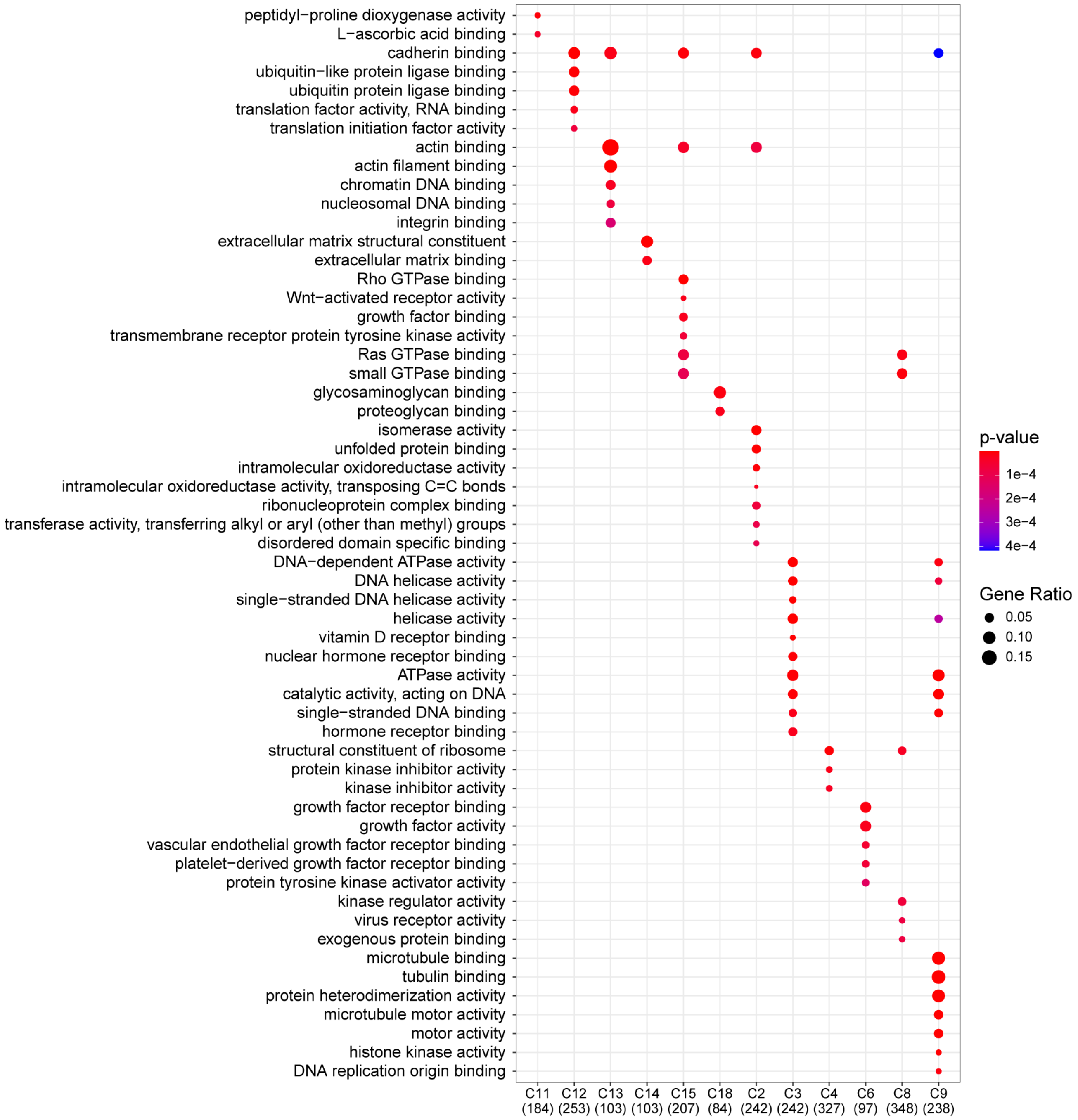

Figure S6. Functional analysis of DEG clusters. The significant GO terms for biological processes for 20 DEG clusters presented in Figure 4b. The x-axis (C#) and the number in parenthesis indicate the cluster ids and the number of DEGs in them.

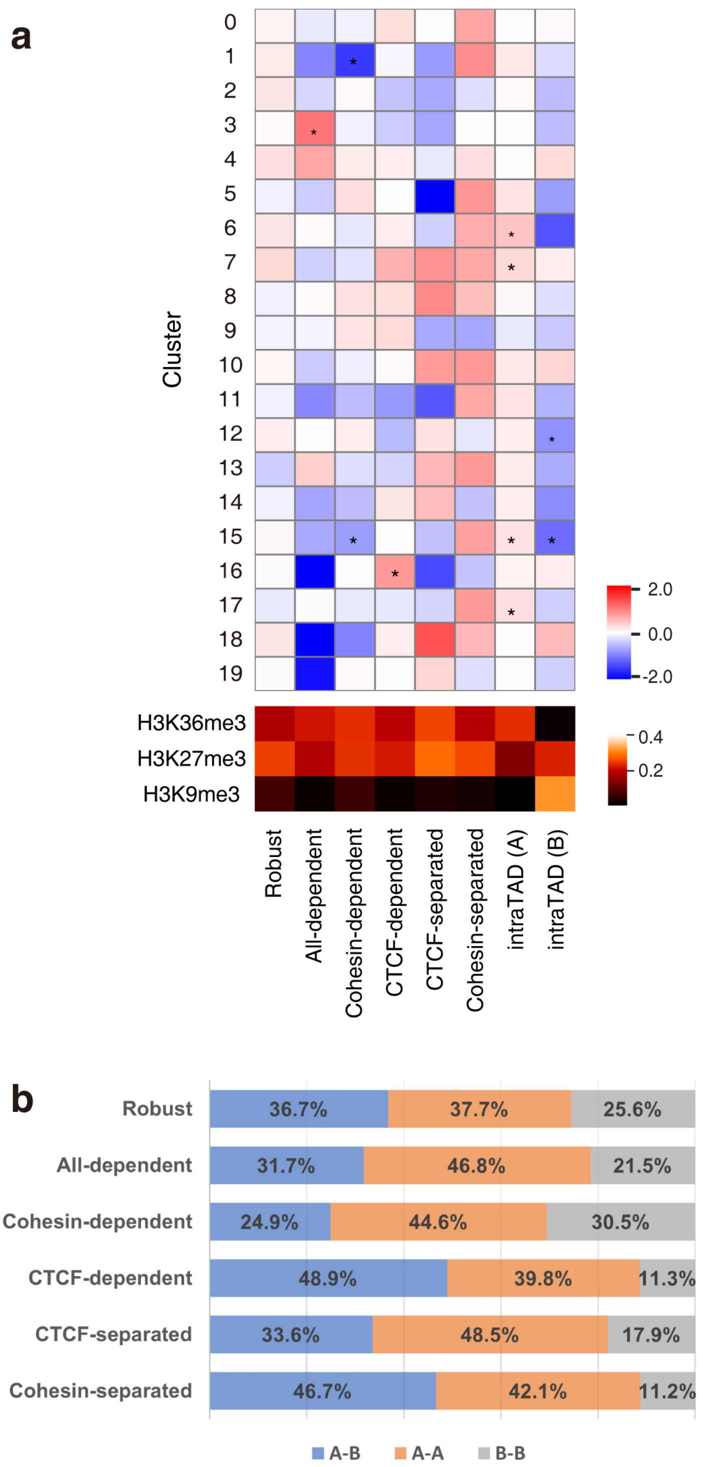

Figure S7. Enrichment of DEGs, histone marks, and compartment boundaries that overlapped the six boundary types. (a) The enrichment of DEGs (top) and broad histone marks (bottom) that overlapped with the six boundary types relative to all boundaries. For the broad histone marks, we calculated the fraction of regions covered by the obtained peaks. *$p < 0.01$ (permutation test, n = 1,000). **(b)** The percentage of identified boundaries located between compartments A and B (A-B) or within compartments A (A-A) and B (B-B).

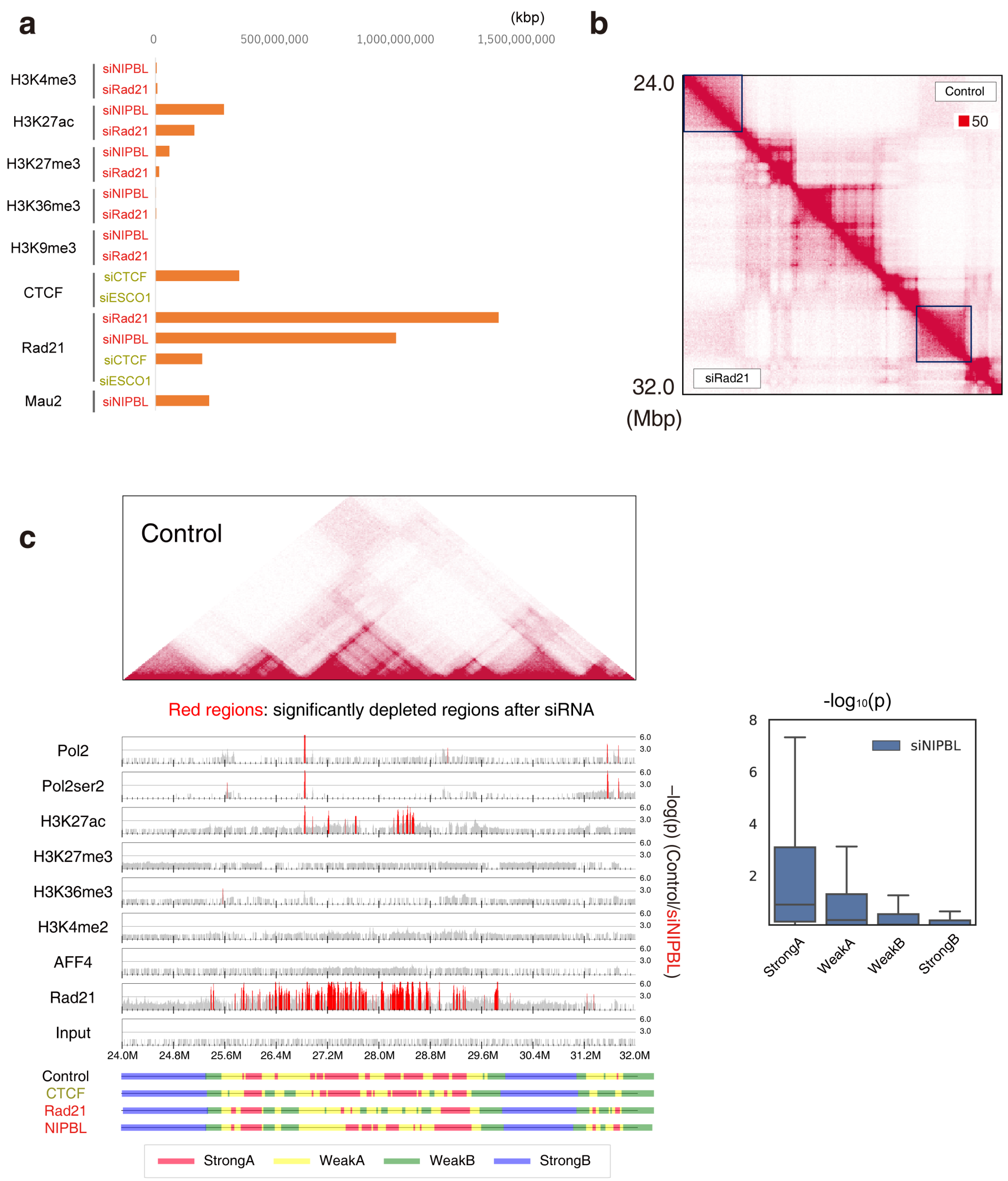

Figure S8. Correlation of depletion effects on epigenome and chromatin folding. (a) The total width of genomic regions in which five histone modifications and ChIP-seq peaks were enriched or depleted for each siRNA target. **(b)** siRad21 had less of an effect on StrongB TADs (black rectangles). This is the same region as shown in Figure 6a. **(c)** Visualization of the biological replicate samples (non spike-in) analogous to Figures 6a (left) and 6b (right).

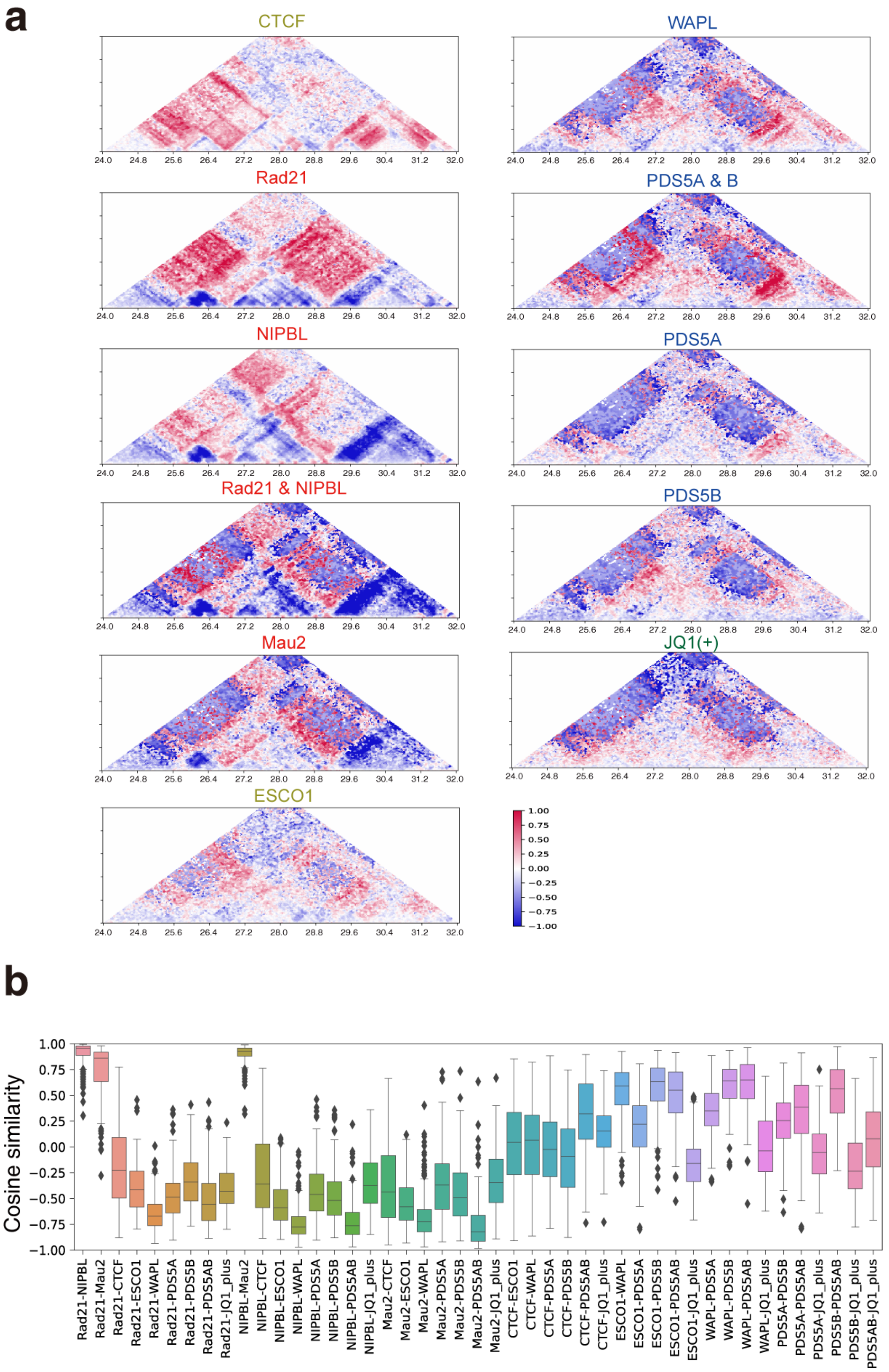

Figure S9. siRNA effects on long-range contact frequency. (a) Relative enrichment of the interaction frequency (log scale) relative to the control for all samples (same chromosomal region as shown in Figure 7a.) **(b)** The cosine similarity distribution of the relative interaction frequency for all 241 differential DRF regions (~2 Mbp from the center of each region).

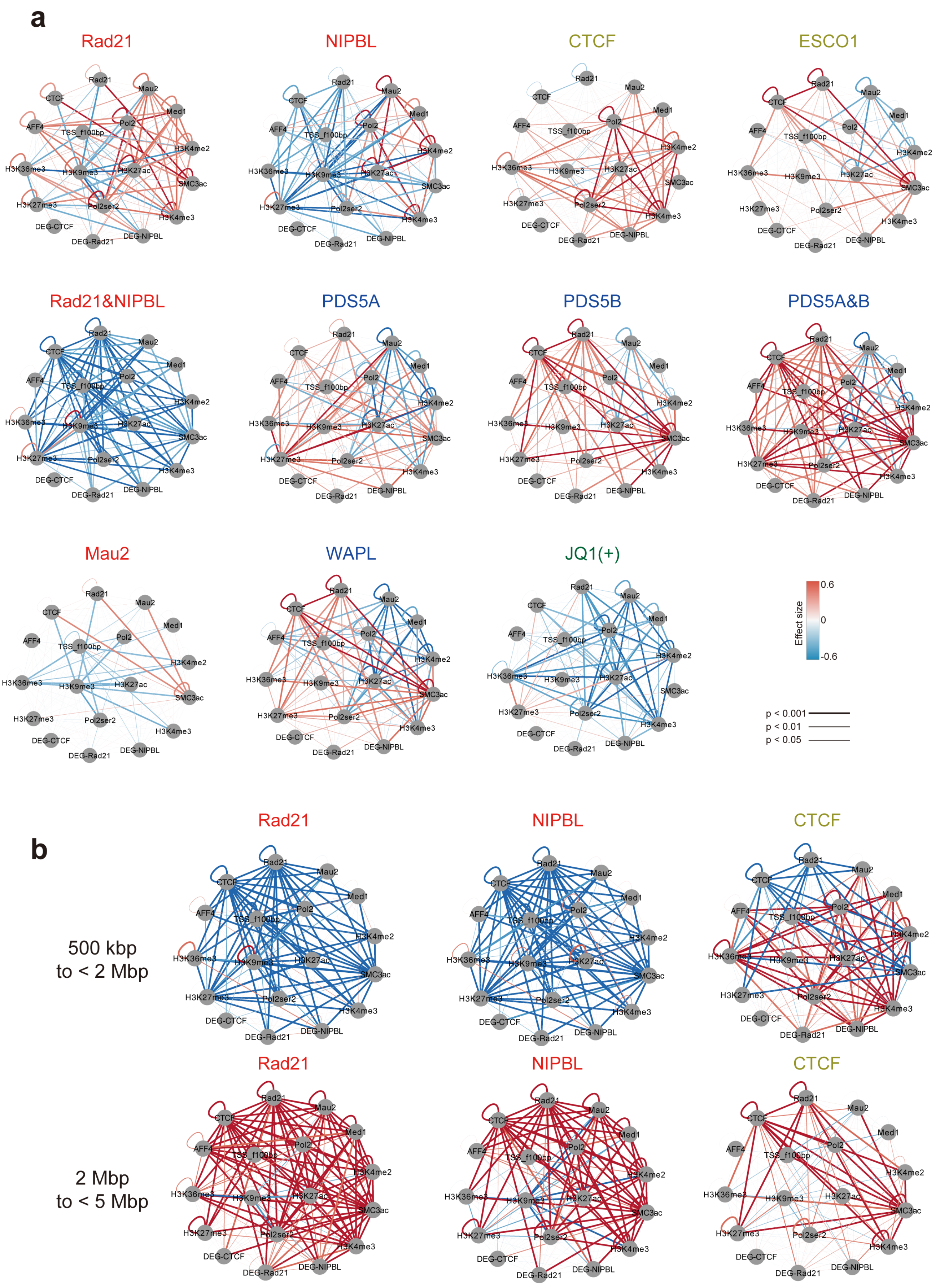

a
Rad21
NIPBL
CTCF
ESCO1
Rad21&NIPBL
PDS5A
PDS5B
PDS5A&B
Mau2
WAPL
JQ1(+)
Rad21
Mau2
Med1
CTCF
AFF4
TSS_f100bp
Pol2
H3K4me2
H3K36me3
H3K9me3
H3K27ac
SMC3ac
H3K27me3
Pol2ser2
H3K4me3
DEG-CTCF
DEG-Rad21
DEG-NIPBL
Effect size
0.6
0
-0.6
p < 0.001
p < 0.01
p < 0.05
b
500 kbp to < 2 Mbp
2 Mbp to < 5 Mbp

Figure S10. SIMA analysis for all samples. (a) SIMA analysis for all samples (distance, 500 kbp–5 Mbp). Color and width of edges correspond to the effect size and significance (Wilcoxon signed-rank test), respectively. **(b)** SIMA analysis for distances of 500 kbp to <2 Mbp (top) and 2 Mbp to <5 Mbp (bottom) for siRad21, siNIPBL, and siCTCF.

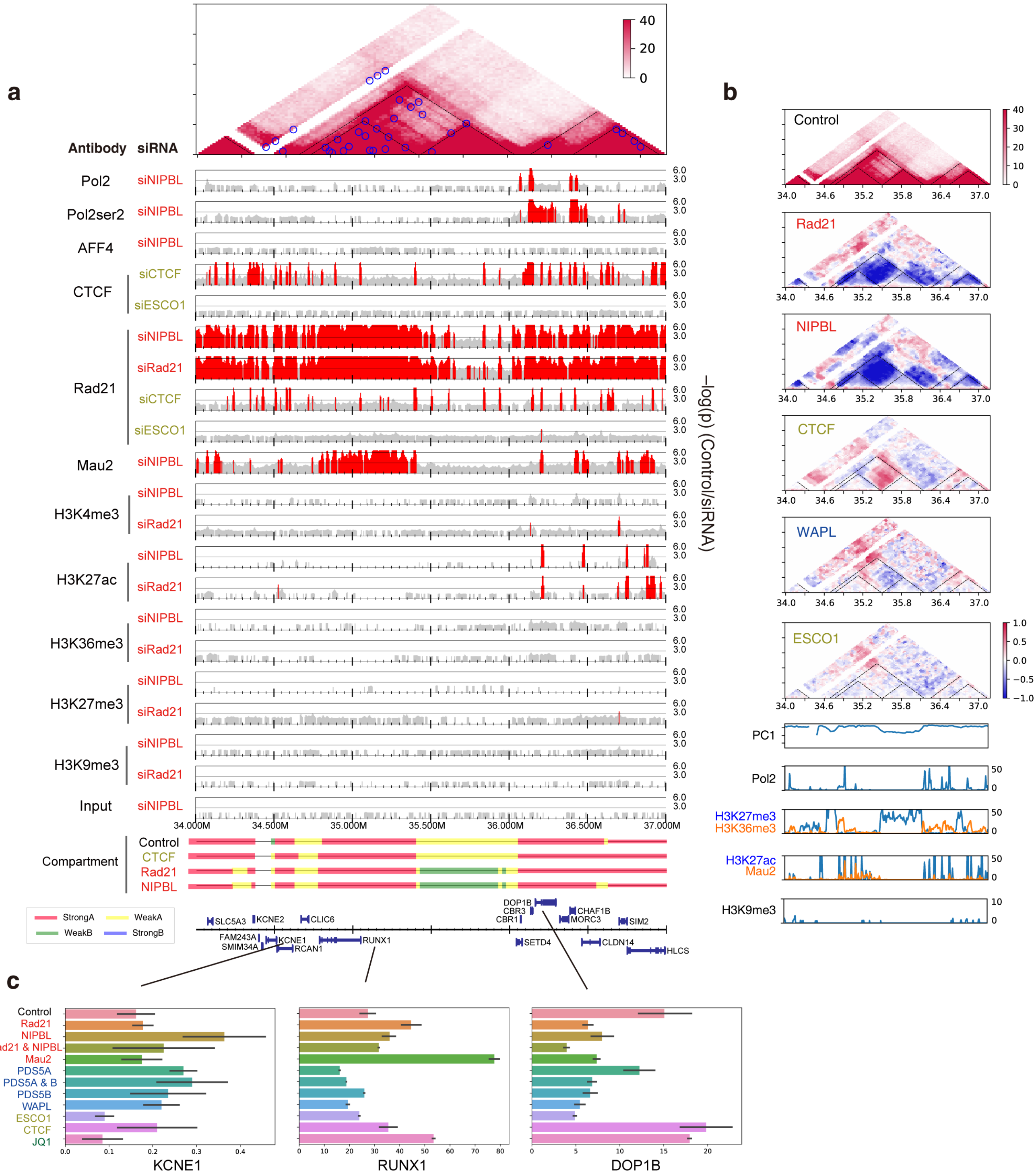

Figure S11. ***RUNX1*** **locus (chromosome 21, 34–37 Mbp.)** **(a)** Top: Hi-C heatmap and the depletion effect distribution of ChIP-seq (–$\log_{10}$(p), control/siRNA, 5-kbp bin.) Black dashed lines and blue circles indicate called TADs and loops, respectively. Middle: The four compartment types as indicated by the colored bars. Bottom: Gene annotation. **(b)** Relative enrichment of interaction frequency and the –$\log_{10}$(p) visualization of ChIP-seq data for the same region. **(c)** Average gene expression level (TPM) for all siRNA targets.
