# Supplementary material for: Context-dependent perturbations in chromatin folding and the transcriptome by cohesin and related factors
Source: Nat Commun. 2023 Sep 19;14:5647. doi: 10.1038/s41467-023-41316-4 (PMC10509244; doi:10.1038/s41467-023-41316-4)
Supplement: Supplementary file 3 — Description of Additional Supplementary Files [file 41467_2023_41316_MOESM3_ESM.pdf]

**Description of Additional Supplementary Files**

File Name: Supplementary Data 1
Description: Statistics and quality metrics of the Hi-C data generated in this study.

File Name: Supplementary Data 2
Description: Statistics and quality metrics of the RNA-seq data generated in this study.

File Name: Supplementary Data 3
Description: Statistics and quality metrics of the ChIP-seq data generated in this study.

File Name: Supplementary Data 4
Description: Twenty clusters of all DEGs.

File Name: Supplementary Data 5
Description: Six categories of all TAD boundaries based on their insulation scores.

File Name: Supplementary Data 6
Description: 241 genomic regions in which DRF values changed significantly after cohesin or loader depletion.

File Name: Supplementary Data 7
Description: The siRNA sequences.
