# Supplementary material for: Context-dependent perturbations in chromatin folding and the transcriptome by cohesin and related factors
Source: Nat Commun. 2023 Sep 19;14:5647. doi: 10.1038/s41467-023-41316-4 (PMC10509244; doi:10.1038/s41467-023-41316-4)
Supplement: Supplementary file 11 — Reporting Summary [file 41467_2023_41316_MOESM11_ESM.pdf]

nature portfolio

Corresponding author(s): Ryuichiro Nakato

Last updated by author(s): Aug 10, 2023

Reporting Summary

Nature Portfolio wishes to improve the reproducibility of the work that we publish. This form provides structure for consistency and transparency in reporting. For further information on Nature Portfolio policies, see our Editorial Policies and the Editorial Policy Checklist.

Statistics

For all statistical analyses, confirm that the following items are present in the figure legend, table legend, main text, or Methods section.

n/a	Confirmed	
☐	☒	The exact sample size (*n*) for each experimental group/condition, given as a discrete number and unit of measurement
☐	☒	A statement on whether measurements were taken from distinct samples or whether the same sample was measured repeatedly
☐	☒	The statistical test(s) used AND whether they are one- or two-sided<br>*Only common tests should be described solely by name; describe more complex techniques in the Methods section.*
☒	☐	A description of all covariates tested
☐	☒	A description of any assumptions or corrections, such as tests of normality and adjustment for multiple comparisons
☐	☒	A full description of the statistical parameters including central tendency (e.g. means) or other basic estimates (e.g. regression coefficient) AND variation (e.g. standard deviation) or associated estimates of uncertainty (e.g. confidence intervals)
☐	☒	For null hypothesis testing, the test statistic (e.g. *F*, *t*, *r*) with confidence intervals, effect sizes, degrees of freedom and *P* value noted<br>*Give P values as exact values whenever suitable.*
☒	☐	For Bayesian analysis, information on the choice of priors and Markov chain Monte Carlo settings
☒	☐	For hierarchical and complex designs, identification of the appropriate level for tests and full reporting of outcomes
☒	☐	Estimates of effect sizes (e.g. Cohen's *d*, Pearson's *r*), indicating how they were calculated

Our web collection on statistics for biologists contains articles on many of the points above.

Software and code

Policy information about availability of computer code

Data collection	The Hi-C, RNA-seq and ChIP-seq data were sequeced by Illumina HiSeq-2500 or X Ten system. The samples and mapping statistics are summarized in Tables S1-S3.
Data analysis	Computational tools used in this study are listed in the METHODS section and below.<br><br>Python 3.7 (https://www.python.org/)<br>ImageJ v1.2.0 (https://imagej.nih.gov/ij/index.html)<br>CustardPy v1.0.0 (https://custardpy.readthedocs.io/)<br>BWA v0.7.17 (http://bio-bwa.sourceforge.net/)<br>Samtools v1.15 (http://samtools.sourceforge.net/)<br>Bowtie2 v2.4.1 (http://bowtie-bio.sourceforge.net/bowtie2/index.shtml)<br>STAR v2.7.3a (https://github.com/alexdobin/STAR)<br>RSEM v1.3.1 (https://github.com/deweylab/RSEM)<br>DESeq2 (https://bioconductor.org/packages/release/bioc/html/DESeq2.html)<br>Juicer v1.5.7, Juicer tools v1.9.9 (https://github.com/aidenlab/juicer)<br>The 4DN Hi-C data processing pipeline v43 (Cooler) (https://github.com/4dn-dcic/docker-4dn-hic)<br>cooltools (https://cooltools.readthedocs.io/)<br>HOMER (http://homer.ucsd.edu/homer)<br>3DChromatin_ReplicateQC (https://github.com/kundajelab/3DChromatin_ReplicateQC)

HiC1Dmetrics v0.2.1 (https://github.com/wangjk321/HiC1Dmetrics)
DROMPAplus v1.12.1 (https://github.com/rnakato/DROMPAplus)
SSP v1.2.2 (https://github.com/rnakato/SSP)
ClusterProfiler v4.0.5 (https://bioconductor.org/packages/release/bioc/html/clusterProfiler.html)
ChromHMM v1.23 (http://compbio.mit.edu/ChromHMM/)
liftOver tool (https://genome-store.ucsc.edu/)
Cytoscape v3.8.2 (https://cytoscape.org/)
Singularity v3.6.4 (https://sylabs.io/singularity)
BEDTools v2.28.0 (https://bedtools.readthedocs.io/en/latest/)

The Custom code for the analysis is available on Zenodo (https://doi.org/10.5281/zenodo.8218447).

For manuscripts utilizing custom algorithms or software that are central to the research but not yet described in published literature, software must be made available to editors and reviewers. We strongly encourage code deposition in a community repository (e.g. GitHub). See the Nature Portfolio guidelines for submitting code & software for further information.

Data

Policy information about availability of data

All manuscripts must include a data availability statement. This statement should provide the following information, where applicable:

- Accession codes, unique identifiers, or web links for publicly available datasets
- A description of any restrictions on data availability
- For clinical datasets or third party data, please ensure that the statement adheres to our policy

The human reference genome hg38 was obtained from the UCSC Genome Browser (https://genome.ucsc.edu/). The raw sequencing data and processed files for the Hi-C, RNA-seq, and ChIP-seq data from this study have been submitted to the Gene Expression Omnibus (GEO) under the accession number GSE196450. The .hic files of the merged Hi-C samples and the reference TAD and loop files are also available on GSE196034. The reference data of TAD and loops obtained from the merged control sample are available on Zenodo (https://doi.org/10.5281/zenodo.8218447).

Human research participants

Policy information about studies involving human research participants and Sex and Gender in Research.

Reporting on sex and gender	not applicable
Population characteristics	not applicable
Recruitment	not applicable
Ethics oversight	not applicable

Note that full information on the approval of the study protocol must also be provided in the manuscript.

Field-specific reporting

Please select the one below that is the best fit for your research. If you are not sure, read the appropriate sections before making your selection.

☒ Life sciences ☐ Behavioural & social sciences ☐ Ecological, evolutionary & environmental sciences

For a reference copy of the document with all sections, see nature.com/documents/nr-reporting-summary-flat.pdf

Life sciences study design

All studies must disclose on these points even when the disclosure is negative.

Sample size	Sample sizes were chosen to provide sufficient material for multi-omics data according to common practice in the field.
Data exclusions	The sample of siNIPBL (24-h treatment) was excluded from the downstream analysis due to the insufficient depletion efficiency.
Replication	RNA-seq analysis was performed in duplicates. The principle samples in Hi-C and ChIP-seq data have two or more replicates. We have confirmed the sufficient similarity among replicates, as shown in Figures 1C and 6E. All blots were repeated for at least two biological replicates. All replicated experiments were successful.
Randomization	Sample randomization is not relevant to this study because we did not use experiment groups.
Blinding	Blinding is not relevant to this study because we did not use experiment groups.

Reporting for specific materials, systems and methods

We require information from authors about some types of materials, experimental systems and methods used in many studies. Here, indicate whether each material, system or method listed is relevant to your study. If you are not sure if a list item applies to your research, read the appropriate section before selecting a response.

Materials & experimental systems

n/a	Involved in the study
☐	☒ Antibodies
☐	☒ Eukaryotic cell lines
☒	☐ Palaeontology and archaeology
☒	☐ Animals and other organisms
☒	☐ Clinical data
☒	☐ Dual use research of concern

Methods

n/a	Involved in the study
☐	☒ ChIP-seq
☒	☐ Flow cytometry
☒	☐ MRI-based neuroimaging

Antibodies

Antibodies used

Antibodies for ChIP-seq:
Mouse monoclonal anti-H3K4me3, MABI, MABI0304, MABI0304
Mouse monoclonal anti-H3K9me3, MABI, MABI0308, MABI0308
Mouse monoclonal anti-H3K27ac, MABI, MABI0309, MABI0309
Rabbit monoclonal anti-H3K27me3, abcam, ab192985, EPR18607
Mouse monoclonal anti-H3K36me3, MABI, MABI0333, MABI0333
Rabbit monoclonal anti-Pol2, Cell Signaling Technology, 14958, D8L4Y
Mouse monoclonal anti-Phospho RNA Polymerase II CTD (Pol2Ser2), MABI, MABI0602, MABI0602

Rabbit polyclonal anti-Rad21, in house, NA. Rabbit polyclonal antibody against Rad21 was generated using the peptides CEEKLPVIRSEEEKVRFERQKA. The antibodies were described in Minamino et al., 2015.
Mouse monoclonal anti-Smc3ac, in house, NA. It was raised against the peptide SLRRVIGAK(Ac)K(Ac)DQYFLDKKMC. An antibody that only recognizes the acetylated form of human Smc3 peptide was selected from more than 200 antibody candidates. The antibody was described in https://www.cell.com/cell/fulltext/S0092-8674(10)01235-3.
Mouse monoclonal anti-ESCO1, in house, NA. Mouse monoclonal antibody against Esco1 was generated using the peptides CEEKLPVIRSEEEKVRFERQKA. The antibodies were described in Minamino et al., 2015.
Rabbit polyclonal anti-NIPBL, BETHYL, A301-779A
Rabbit polyclonal anti-Mau2, abcam, ab46906
Rabbit polyclonal anti-CTCF, Merck, 07-729
Rabbit polyclonal anti-BRD4, BETHYL, A301-985A50
Rabbit polyclonal anti-AFF4, BETHYL, A302-538A

Antibodies for immunoblotting:
Mouse monoclonal anti-NIPBL, Santa Cruz Biotechnology, sc-374625, C-9
Mouse monoclonal anti-a-tubulin, Merck, T6074, B-5-1-2
Rabbit polyclonal anti-WAPL, Proteintech, 16370-1-AP
Rabbit polyclonal anti-PDS5A, BETHYL, A300-088A
Rabbit polyclonal anti-PDS5B, abcam, ab70299
Rabbit monoclonal anti-CTCF, Cell Signaling Technology, 3417, D1A7
Mouse monoclonal anti-Mau2, This study, NA

Validation

Antibodies for ChIP-seq:
For antibodies against histone modifications, product information is available at: http://web.archive.org/web/20211016023415/https://www.takarabio.com/products/antibodies-and-elisa/primary-antibodies-and-elisas-by-research-area/epigenetic-antibodies/histone

Anti-Pol2 product information is available at: https://www.cellsignal.com/products/primary-antibodies/rpb1-ntd-d8l4y-rabbit-mab/14958

Anti-NIPBL product information is available at: https://www.thermofisher.com/antibody/product/NIPBL-Antibody-Polyclonal/A301-779A

Anti-Mau2 product information is available at: https://www.abcam.com/products/primary-antibodies/scc4-antibody-ab46906.html

Anti-CTCF product information is available at: https://www.merckmillipore.com/SE/en/product/Anti-CTCF-Antibody,MM_NF-07-729?ReferrerURL=https%3A%2F%2Fwww.google.com%2F

Anti-BRD4 product information is available at: https://www.thermofisher.com/antibody/product/BRD4-Antibody-Polyclonal/A301-985A50

Anti-AFF4 product information is available at: https://www.thermofisher.com/antibody/product/MCEF-Antibody-Polyclonal/A302-538A

Anti-Smc3ac was described in: https://www.cell.com/cell/fulltext/S0092-8674(10)01235-3

Anti-Rad21 and anti-ESCO1 were described in Minamino et al., 2015.

Antibodies for immunoblotting:
Anti-NIPBL product information is available at: https://www.scbt.com/p/nipbl-antibody-c-9

Anti-a-tubulin product information is available at: https://www.sigmaaldrich.com/US/en/product/sigma/t6074

Anti-WAPL product information is available at: https://www.ptglab.com/products/WAPAL-Antibody-16370-1-AP.htm

Anti-PDS5A product information is available at: https://www.fortislife.com/products/primary-antibodies/rabbit-anti-scc-112-antibody/BETHYL-A300-088

Anti-PDS5B product information is available at: https://www.abcam.com/products/primary-antibodies/as3-antibody-ab70299.html

Anti-CTCF product information is available at: https://www.cellsignal.jp/products/primary-antibodies/ctcf-d1a7-xp-rabbit-mab/3417

Anti-Mau2 was validated by western blot with or without MAU2 depletion in human culture cells. Please also see Fig. 2b.

Eukaryotic cell lines

Policy information about cell lines and Sex and Gender in Research

Cell line source(s)	RPE and C2C12 cells were purchased from American Type Culture Collection (ATCC) and the European Collection of Authenticated Cell Cultures (ECACC), respectively. Both cells were cultured in DMEM (Wako) supplemented with Penicillin-Streptomycin-L-Glutamine Solution (Wako), 10% fetal bovine serum (Biosera), and 20 mM HEPES-KOH (pH 7.4).
Authentication	None of the cell line was authenticated.
Mycoplasma contamination	The cell line was negative for mycoplasma contamination.
Commonly misidentified lines (See ICLAC register)	No commonly misidentified cell line was used.

ChIP-seq

Data deposition

☒ Confirm that both raw and final processed data have been deposited in a public database such as GEO.

☒ Confirm that you have deposited or provided access to graph files (e.g. BED files) for the called peaks.

Data access links *May remain private before publication.*	https://www.ncbi.nlm.nih.gov/geo/query/acc.cgi?acc=GSE196450
Files in database submission	FASTQ, BED (peaks) and bigWig files.
Genome browser session (e.g. UCSC)	We do not use a genome browser session.

Methodology

Replicates	Rad21 and CTCF ChIP-seq for control cells were performed twice. Because siNIPBL and siRad21 showed closely similar results, ChIP-seq samples under siNIPBL and siRad21 can be considered replicates. Input samples were generated in every experiment. See Supplementary Data 3 for the detail.
Sequencing depth	The sequence reads range from 25 million to 75 million (58 million on average).
Antibodies	Antibodies for ChIP-seq:<br>Mouse monoclonal anti-H3K4me3, MABI, MABI0304<br>Mouse monoclonal anti-H3K9me3, MABI, MABI0308<br>Mouse monoclonal anti-H3K27ac, MABI, MABI0309<br>Rabbit monoclonal anti-H3K27me3, abcam, ab192985<br>Mouse monoclonal anti-H3K36me3, MABI, MABI0333<br>Rabbit monoclonal anti-Rol2, Cell Signaling Technology, 14958

	Rabbit monoclonal anti-Rpb1 NTD, Cell Signaling Technology, 14985<br>Rabbit polyclonal anti-Rad21, Minamino et al., 2015<br>Mouse monoclonal anti-Smc3ac, Minamino et al., 2015<br>Mouse monoclonal anti-ESCO1, Minamino et al., 2015<br>Rabbit polyclonal anti-NIPBL, BETHYL, A301-779A<br>Rabbit polyclonal anti-Mau2, abcam, ab46906<br>Rabbit polyclonal anti-CTCF, Merck, 07-729<br>Rabbit polyclonal anti-BRD4, BETHYL, A301-985A50<br>Rabbit polyclonal anti-AFF4, BETHYL, A302-538A
Peak calling parameters	DROMPAplus v1.12.1 (https://github.com/rnakato/DROMPAplus), 100-bp bin, with spike-in normalization (see METHODS section).<br>"--pthre_internal 1 --pthre_enrich 2" for H3K9me3, "--pthre_internal 5, --pthre_enrich 4" for the others.
Data quality	The quality of ChIP-seq data was evaluated using mapping ratio, read depth, read complexity, GC bias, normalized strand coefficient (NSC), and background uniformity. The result is summarized in Supplementary Data 3.
Software	Bowtie2 v2.4.1 (http://bowtie-bio.sourceforge.net/bowtie2/index.shtml)<br>Samtools v1.15 (http://www.htslib.org/)<br>DROMPAplus v1.12.1 (https://github.com/rnakato/DROMPAplus)<br>SSP v1.2.2 (https://github.com/rnakato/SSP)<br>ChromHMM v1.23 (http://compbio.mit.edu/ChromHMM/)
